# ADV at the Time of COVID-19 Brain Effect between Emotional Engagement and Purchase Intention

**DOI:** 10.3390/brainsci12050593

**Published:** 2022-05-03

**Authors:** Martina Sansone, Michela Balconi

**Affiliations:** 1 International Research Center for Cognitive Applied Neuroscience (IrcCAN), Università Cattolica del Sacro Cuore, 20123 Milan, Italy; michela.balconi@unicatt.it; 2 Research Unit in Affective and Social Neuroscience, Department of Psychology, Università Cattolica del Sacro Cuore, 20123 Milan, Italy

**Keywords:** emotions, purchase intention, brand engagement, consumer neuroscience, fNIRS

## Abstract

In pandemic times, taking advantage of COVID-19-elicited emotions in commercials has been a popular tactic employed by corporations to build successful consumer engagement and, hopefully, increase sales. The present study investigates whether COVID-19-related emotional communication affects the consumer’s emotional response and the approach/avoidance motivation toward the brand—measured as a function of brain hemodynamic changes—as well as the purchase intentions. The functional Near-Infrared Spectroscopy (fNIRS) was employed to record neural correlates from the prefrontal cortex while the experimental and control groups were observing respectively COVID-19-related and unrelated advertisements (ads). The hemodynamic patterns suggest that COVID-19-related ads may promote deeper emotional elaboration, shifting consumers’ attention from the semantic meaning to the affective features and perhaps supporting a more favorable brand evaluation. Conversely, purchase intentions were only related to the pre-existing level of brand engagement. The findings suggest that leveraging the negative emotional potential of COVID-19 may not shift the explicit purchase intentions but could nonetheless boost emotional engagement, benefitting the final evaluation of the brand at an implicit level.

## 1. Introduction

As the COVID-19 pandemic goes towards new stages two years after its outbreak, consumers continue to keep find new ways to adapt their habits to new rules and limitations, passing through lockdowns and attempts of going back to a “new normal”. It is evident that not only has the pandemic affected the consumer journey from a physical and logistical perspective, but it has profoundly affected individuals on a psychological level, too. By now, numerous studies have warned against the alarming consequences of the pandemic on several psychological and social aspects, such as the increase in anxiety and depressive symptomatology, the worsening of the quality of life, and financial hardship [1,2,3]. The outbreak of COVID-19 disrupted the perception of ontological security and generated uncertainty and fear about the contagion threat [4]. As it generally occurs for natural disasters and extraordinary events that change the world [5], the threat perception has pushed the population to adopt self-protective coping strategies in an attempt to stay safe, to reduce the contagion risk [6], and to re-establish a sense of control [7], also resulting in profound modifications in purchase patterns and consumption behaviors [8,9,10].

As a matter of fact, during the early stages of the pandemic, we witnessed panic buying behaviors such as hoarding, which is a frequent reaction to the uncertainty of future supplies of fundamental necessities [4,11,12]. When consumers are reminded of the risks of COVID-19 illness, the consumer preference may shift towards products that respond to their desire for psychological safety and control, such as more familiar or authentic products, which are considered more genuine and reliable [7,13]. On the other hand, hints of infection may also drive consumers to adopt less common behaviors, such as preferring unusual items—because their mental representation is implicitly linked with fewer persons, suggesting a reduced potential for virus transmission [14]—or even to increase variety-seeking in their purchases in an attempt to regain control, freedom, and self-determination. Becoming aware of the threat posed by COVID-19 to one’s health was also shown to increase the intention to purchase products that reduce health risks, such as organic food [15] and indoor fitness products [16]. Stress and anxiety generated by the pandemic indeed can prompt a greater engagement with health- and COVID-19-related products and advertisements, which are made salient to individuals by the public health crisis. Such thorough processing of the message, motivated by COVID-19-related stress, predicts a more positive attitude toward the advertisement, which in turn increases the intention to purchase the advertised product [16].

In this regard, during the pandemic, it seems that many corporations have intuitively recognized the possibility of exploiting the COVID-19 reference in commercials as a source of customer engagement to promote emotional reactions and favorable attitudes toward the brand [17]. Notably, including the consumer attitude toward the advertisement and the affective response into the equation is a crucial component for the understanding of consumer behavior. Many scholars have collected evidence that the attitude—that represents a favorable or unfavorable summary appraisal of the ad [18]—can be predictive of consumer choices [19,20] and indicative of the advertisement‘s effectiveness [21].

Consumer neuroscience studies have further suggested that an effective way to measure the consumer’s favorable or unfavorable response is to assess the balance of the neural activity within the prefrontal cortex (PFC), namely, the Frontal Asymmetry Index (FAI). Indeed, the PFC was shown to host the neural correlates of the two motivational systems that are thought to control human behavior [22]. Specifically, a prevalence of left frontal activity is indicative of approach dispositions and appetitive behavioral tendencies, whereas a prevalence of right frontal activity is indicative of avoidance dispositions and withdrawal behavioral tendencies [23,24]. Because of the relationship that links PFC and motivational attitudes, the prefrontal activity is generally sensitive to consumer preferences and attitudes toward the advertised content [25,26,27] and is considered a predictor of purchase intention [28]. For instance, Ravaja et al. [29] found that left-lateralized frontal activity was associated with increased purchase decisions when observing images of items varying in brand and price. One further crucial factor to consider in the study of advertising effectiveness is that feelings and emotional responses to the ad are closely involved in attitude formation [30,31] and exert a strong influence on consumer behavioral intentions (such as purchase intention) both directly and through the intermediary variables of the attitude toward the advertisement and the brand [18,20,32]. Specifically, it has been argued that advertisements that leverage emotional content are more effective than rational appeals in promoting purchase intention [33], a notion that probably contributed to spreading the practice of resorting to intense, emotionally charged ads.

However, scholars have long been divided on the effectiveness of emotional advertising. On the one hand, it is commonly accepted that positive emotional contents are more effective than negative contents for persuasive purposes [34,35]. However, there is evidence that negative emotions can be even more effective than positive emotions on some occasions (see [36] for a review). Crucially, despite being a very common practice, heavy reliance on emotions could be a particularly risky strategy in times of pandemic. Indeed, during such a harsh time, displaying intense emotions (especially negative ones) could trigger aversive responses, such as anxiety or irritation [37,38]. This could further set off unfavorable attitudes toward the ad and the brand itself, weakening the purchase intention [18,33]. Yet, since the pandemic outbreak, employing emotional commercials openly referring to COVID-19 (a disrupting event that changed our lives in a negative way all around the world) has been a popular tactic for firms to sell their brand image and connect with consumers more successfully [17]. Prior studies [39] have offered preliminary evidence about the efficacy of COVID-19-related emotional ads on emotional engagement; however, the effect on concrete behavioral intentions was not investigated. Given the conflicting findings on negative emotions in ads, the goal of the present study is to shed some light on the impact of COVID-19 information on consumer behavior, in an effort to understand whether the vivid emotions elicited by the COVID-19 stimuli can affect the emotional responses, the attitudes toward the brand, and, ultimately, the intention to buy products from the advertised brand.

Based on previous evidence, to assess participants’ dispositions toward the advertisements that were displayed in the present study, neural activity from PFC was recorded with the functional Near-Infrared Spectroscopy (fNIRS), a solid, non-invasive tool that allows assessing the brain’s neural activity by monitoring variations in the cortical hemoglobin concentration with accurate spatial and temporal resolution [40]. The fNIRS can provide accurate esteem of lateralized prefrontal activity [41,42] and has recently proven its potential also in consumer neuroscience research [43,44,45].

First, building on the evidence that the PFC is part of a well-established neural network involved in the processing of emotional stimuli [46,47], we hypothesized that COVID-19 related appeals would trigger more activity within the PFC compared to appeals nonrelated to COVID-19 because we expect the first to elicit stronger emotional responses. Secondly, following the prefrontal asymmetry theory [24], we hypothesized finding left prefrontal dominance in response to ads that prompt a favorable evaluation and approach tendencies, and right dominance for ads that prompt an unfavorable evaluation and avoidance (irrespective of the valence), as measured by the hemodynamic changes recorded in the PFC with the fNIRS. Moreover, we expected to find a correlation between the PFC neural activity and participants’ explicit intention to purchase from the advertised brand, measured with the Purchase Intention scale [18], and that such correlation would differ in the two groups if one ad type was more effective than the other. Finally, a similar effect was checked in relationship with the participants’ preexisting preference for the brand displayed during the experiment in order to assess whether eventual differences in the purchase intention measure of the two groups (COVID-19 vs. non-COVID-19) could be explained by the level of brand engagement.

## 2. Materials and Methods

### 2.1. Subjects

A total of 19 healthy Italian participants, ranging from 20 to 28 years old (M age = 25.03; SD age = 2.04; 5 men) took part in the experiment. Exclusion criteria involved the presence of any psychopathological or neurological disorder, acute medical conditions, head trauma, or ongoing psychopharmacological treatment. In addition, high levels of post-traumatic stress symptoms related to the COVID-19 experience, exceeding the COVID-19-PTSD questionnaire cut-off [48], would cause the exclusion of the participant from the study. All participants had normal-to-corrected eyesight and were right-handed; they were mainly graduate and undergraduate students selected from the Catholic University of the Sacred Heart of Milan’s university campus, and they all gave written consent prior to their participation. No monetary compensation was provided for their involvement in the study. Participants were randomly assigned to the experimental condition, which required them to watch COVID-19-related advertising, or to the control condition, requiring them to watch COVID-19-unrelated advertising. The COVID-19 group (M = 26.87, SD = 1.34) and non-COVID-19 group (M = 25.78, SD = 1.90), were age-matched. The experiment took place between July and September 2021, when Italy was experiencing a mild relaxation of the COVID-19 safety measures, one year after the pandemic outbreak. The research protocol was carried out in accordance with the Declaration of Helsinki (1964) and was approved by the ethics committee of the Catholic University of the Sacred Heart of Milan’s Department of Psychology.

### 2.2. Advertising Stimuli

Because the study sought to understand the effect of COVID-19 cues on advertising communication, the stimuli were selected with a view to confronting advertisements that only differ in the presence/absence of COVID-19-related information, while presenting comparable emotional and motivational tones. Most COVID-19-related advertisements produced during the pandemic adopted an empowering tone of voice [17]. Among the brands that have produced COVID-19-related advertisements, Nike was selected, as its communication is traditionally shaped by an empowering tone of voice, and it is considered a trademark of the brand. Hence, the stimulus set comprised six advertising spots by Nike, a famous sportswear brand with a strong connotation in terms of corporate social responsibility, social advocacy, and empowering communication.

“Play for the World”, “You Can’t Stop LA”, and “You Can’t Stop Us” are three ads that allude to the COVID-19 theme chosen for the study. Images presenting a play of references to the current harsh historical time, with clear cues hinting at the pandemic, are combined with the brand’s usual communication, which is rich in emotional and inspirational aspects. 

The “Play for the World” commercial’s plot refers to the scenario of the first 2020 lockdown, with individuals being compelled to stay at home. Ordinary people and famous athletes practice within the walls of their homes or on their driveways because of the restrictions imposed by the lockdown, and their images take turns on the screen in slow motion together with images of empty playgrounds. The commercial “You Can’t Stop LA” depicts the wins and losses of the Los Angeles Lakers basketball club, which are alternated with poignant episodes of 2020, depicting an analogy with the societal accomplishments and defeats collected in the human match fought against the pandemic. The storyline of “You Can’t Stop Us” features an encouraging message that focuses on the narrative of the world’s comeback from the lockdown. The split-screen technique is used to create powerful images made up of the coupling of different sporting events scenes, as well as empty stadiums and people finding alternative ways to train at home. The return to live sports competitions after the end of the lockdown is stressed in the end.

“What’s your motivation?”, “You can’t be stopped”, and “Steps” are the three selected advertisements that do not recur to COVID-19-related information; nevertheless, they convey emotional and motivating components that typically qualify Nike’s communication. “What’s your motivation?” tells of a young basketball player who trains hard and reflects on success, pointing out that success does not happen by accident but rather demands a lot of practice. The commercial “You Can’t Be Stopped” aims to urge athletes in uncovering the deep motivations that drive us, reminding the audience that when we pursue our true motivation, we cannot be stopped. Finally, “Steps” depicts with an inspirational tone the story of a runner’s journey through challenges and failures.

All the stimuli were previously validated in a preliminary study [39].

### 2.3. Questionnaires

#### 2.3.1. COVID-19-PTSD Questionnaire

The COVID-19-PTSD questionnaire [48] was administered prior to the experimental session to control for the presence of Post-Traumatic Stress Disorder symptoms. The questionnaire is composed of 7 sub-scales investigating Intrusion, Avoidance, Negative Affect, Anhedonia, Dysphoric arousal, Anxious arousal, and Externalizing behavior. The questionnaire comprises 19 items that require subjects to respond on a 5-point Likert scale, from 0 (not at all) to 4 (extremely). Scores for each item were summed to give a total score of COVID-19-related PTSD symptomatology. The range of possible total scores was 0–76, with higher scores indicating higher levels of PTSD symptoms. The cut-off was set at 33, with values equal to or higher than 33 indicating a high risk of PTSD symptomatology. In our sample, the total scores ranged from 0 to 28 (*M* = 10.55; *SD* = 8.12).

#### 2.3.2. Brand Engagement in Self-Concept Questionnaire (BESC)

A modified version of the Brand Engagement in Self-Concept (BESC) [49] was used to control for the preexisting level of engagement with the brand advertised by the stimuli. The BESC is a single-scale questionnaire that assesses the tendency to include brands in the self-concept, capturing the general engagement of an individual with brands. The questionnaire was adapted specifically to the Nike brand to measure the level of engagement with the brand shown in the experimental manipulation. The modified scale comprises 8 statements, such as “I often feel a personal connection with the Nike brand”. Participants were to rate their degree of agreement on a 7-step Likert scale (1 = “strongly disagree”, 7 = “strongly agree”). Scores for each item were summed to give a total score of brand engagement with Nike. The range of possible total scores was 8–56, with higher scores indicating higher levels of engagement. In our sample, the total scores ranged from 8 to 34.

#### 2.3.3. Purchase Intention Scale (PI)

The purchase Intention scale (PI) [18] was used to investigate the participants’ personal action tendencies with respect to the brand, and their motivation to exert effort to carry out the purchase behavior. The scale was adapted to the Nike brand. Participants were asked to describe their overall intentions about the brand advertised during the experiment with a seven-point semantic differential. The modified scale comprises 5 items, such as “definitely do not intend to buy/definitely intend to buy”. Scores for each item were summed to give a total score of purchase intention. The range of possible total scores was 5–35, with higher scores indicating higher levels of intention to purchase. In our sample, the total scores ranged from 13 to 32.

### 2.4. Procedure

**Figure 1:**
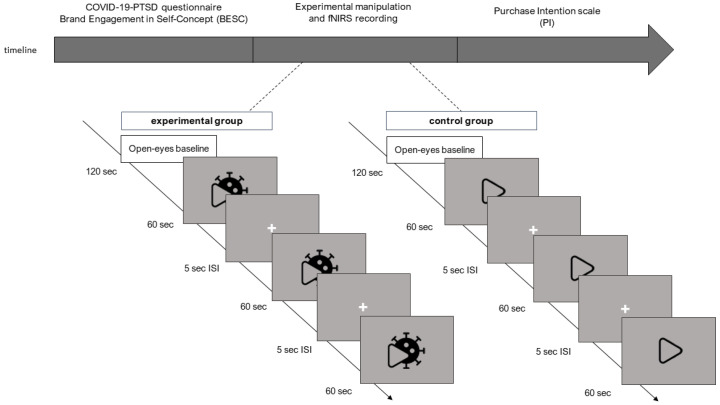
Experimental procedure. The timeline of the study is shown in Figure 1. After the COVID-19-PTSD questionnaire and the Brand Engagement in Self-Concept (BESC) scale were administered, a 120 s open-eyes baseline was recorded with the fNIRS (functional Near-Infrared Spectroscopy). The neural correlates were recorded during the experimental manipulation, which differed between the two groups: the experimental group (on the left) observed the three COVID-19-related video ads, while the control group observed the three COVID-19-unrelated video ads (on the right), in randomized order. Each stimulus lasted 60 s. and an inter-stimulus interval of 5 s was displayed before the next stimulus. Finally, the Purchase Intention (PI) scale was filled out.

The participants comfortably sat on a chair in a dimly lit room in front of a computer monitor that was placed about 80 cm from the subject. Prior to the experiment, the participants were to compile the modified version of the Brand Engagement in Self-Concept (BESC) [49]. At the start of the experiment, the resting hemodynamic activity was measured with a 120-s open-eyes baseline. Following the baseline, the experimental group was shown the three COVID-19-related commercials, while the control group was shown the non-COVID-19-related commercials. The ads were exhibited in randomized sequence at the center of the screen, and a 5-s inter-stimulus-interval was displayed between the commercials, during which a black screen was presented. During the ad viewing, hemodynamic changes were recorded with the fNIRS. At the end of the experimental session, the participants were asked to complete the Purchase Intention scale (PI) [18]. The entire experiment took about 25 min to complete (Figure 1).

### 2.5. fNIRS Configuration

**Figure 2:**
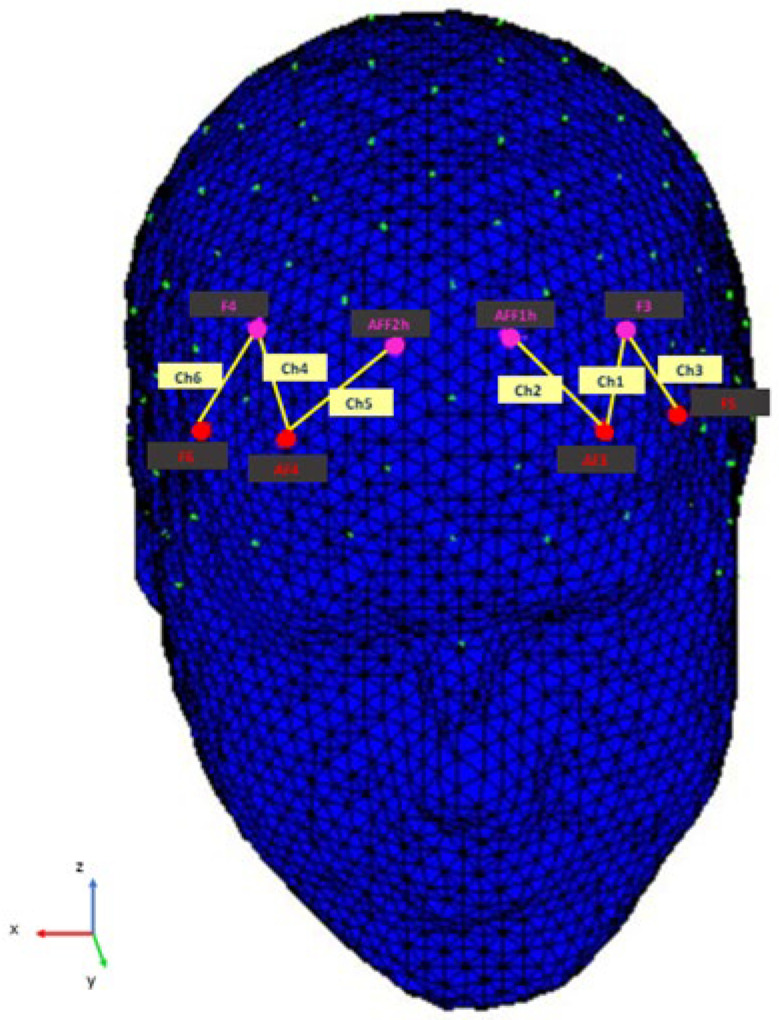
Configuration of fNIRS probes. The sources were placed in AF3–AF4 and F5–F6 (red), while the detectors were placed in AFF1h–AFF2h and F3–F4 (purple). The six resulting channels (yellow) were meant to detect the neural activity from the following areas: Ch1 and Ch4 from the left and right DLPFC (BA 46); Ch2 and Ch5 from the left and right frontopolar area (BA10) and a portion of left and right DLPFC (BA 46); Ch3 and Ch6 from the left and right Broca’s area pars triangularis (BA45). The 3D head render was created with the software nirsLAB (NIRx Medical Technologies LLC), and then the corresponding channels were added.

The hemodynamic signal was captured with the NIRScout System (NIRx Medical Technologies, LLC, Los Angeles, California). A 6-channel optodes matrix was used to measure oxygenated hemoglobin (O2Hb) and deoxygenated hemoglobin (HHb) concentration changes over the prefrontal area. The optodes were mounted on the subject’s head using an fNIRS Cap that complies with the conventional international 10/5 system [50]. Specifically, four emitters were mounted in AF3-AF4, F5-F6, and four detectors were mounted in AFF1h-AFF2h, F3-F4. For contiguous optodes, the emitter-detector distance was 30 mm, and two wavelengths of near-infrared light were employed (760 and 850 nM). The optode configuration resulted in a total of six channels, formed as follows: Ch1 (AF3-F3), Ch2 (AF3-AFF1h), Ch3 (F5-F3), Ch4 (AF4-F4), Ch5 (AF4-AFF2h), and Ch6 (F6-F4) [51,52]. The automated anatomical labeling atlas Brodmann, included in the software fOLD (fNIRS Optodes’ Location Decider) [53], was used to identify with a probabilistic approach the specific cortical areas responsible for the hemodynamic modifications observed during the task [54]. The fNIRS channels-Brodmann areas match identified by the software is as follows: Ch1 and Ch4 are consistent with left and right DLPFC (BA 46); Ch2 and Ch5 match with the left and right frontopolar area (BA 10), as well as a portion of left and right DLPFC (BA 46); Ch3 and Ch6 cover the left and right pars triangularis within the Broca’s area (BA 45) (Figure 2).

### 2.6. fNIRS Biosignal Analysis

The variations in O2Hb and HHb were measured continuously with NIRStar Acquisition Software during the 120-s open-eyes baseline at rest and throughout the task. The signal was collected at a sampling rate of 6.25 Hz. NirsLAB software (v2014.05; NIRx Medical Technologies LLC, Glen Head, NY, USA) was adopted to analyze the biosignals according to their wavelength and position, producing mmoL * mm values corresponding to changes in O2Hb and HHb concentrations per channel. The biosignals received from each channel were filtered using a digital band-pass filter at 0.01–0.3 Hz to exclude artifacts. For each channel, the O2Hb and HHb values were averaged, and the effect size was calculated in each condition using the average concentrations in the time series for each channel and individual. The effect sizes (Cohen’s d values) were calculated using the formula D = (m1 − m2)/s, where m1 and m2 indicate the baseline and trial mean concentration levels, respectively, and s represents the baseline SD. To increase the signal-to-noise ratio, the effect sizes from the six channels were averaged. Because the effect size is unaffected by the differential pathlength factor (DPF), it was possible to average the normalized effect size data regardless of the unit. The same averaging procedure would not have been possible on raw fNIRS data, which were originally relative values that could not be directly averaged across subjects or channels.

### 2.7. Statistical Data Analysis

Two repeated-measures ANOVAs were performed on D-dependent fNIRS data (O2Hb and HHb mean values) using Group (2: COVID-19, non-COVID-19) as independent between factor and Channel (6: Ch1, Ch2, Ch3, Ch4, Ch5, Ch6) as independent within factor [55,56]. Pairwise comparisons were used to check simple effects for significant interactions, and Bonferroni correction was employed to reduce biases in repeated comparisons. For all ANOVAs, the Greenhouse–Geisser epsilon was employed to adjust the degrees of freedom. To determine the normality of the data distribution, the kurtosis and asymmetry indices were examined. The size of statistically significant effects has been estimated using partial eta squared (η^2^) indices.

Secondly, a set of correlational analyses was conducted to investigate the relationship between the prefrontal activity and purchase intention; the prefrontal activity and brand engagement; purchase intention and brand engagement, considering both the two groups separately and then the whole sample.

## 3. Results

**Figure 3:**
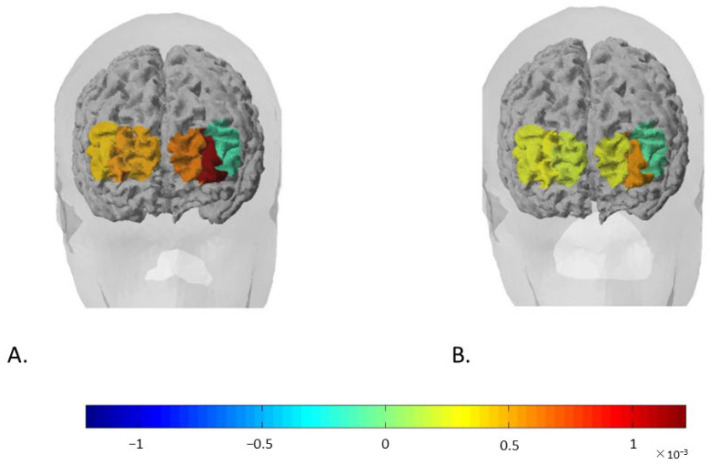
Rendering representation of cortical activity. The cortical maps show the modifications of O2Hb levels as a function of the type of commercials viewed by (**A**) the experimental group (COVID-19-related ads) and (**B**) the control group (ads unrelated to COVID-19).

The ANOVA performed on the D-dependent measures for O2Hb mean values revealed a significant main effect of the Group (F [2,18] = 7.76, *p* = 0.01, η^2^ = 0.398), with participants displaying higher mean values in the COVID-19-related condition compared to the non-COVID-19 condition (Figure 3).

Finally, a significant interaction effect between Group and Channel was found (F [5,18] = 7.08, *p* = 0.01, η^2^ = 0.395). Pairwise comparisons revealed that the D values in Channel 1 were significantly higher than those in Channel 3 only in the COVID-19-related condition (F [1,18] = 9.77, *p* = 0.01, η^2^ = 0.498) (Figure 3).

Conversely, statistical analysis for the HHb mean values yielded no significant effects.

**Figure 4:**
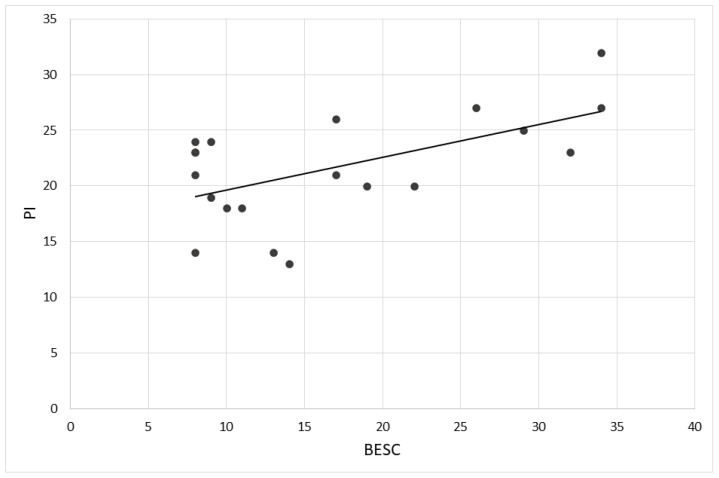
Correlation between Purchase Intention and Brand Engagement in self-concept. The graph represents the positive correlation between the Purchase Intention scale (PI) and the Brand Engagement in Self-Concept (BESC) scores found across all participants (*r* = 0.576, *p* = 0.008).

About the correlational analysis, firstly, the brand engagement and the intention to purchase did not correlate when considering the two groups individually (COVID: *r* = −0.173, *p* > 0.5); non-COVID: *r* = 0.623, *p* = 0.54). However, a positive correlation was found between brand engagement and purchase intention only when taking into account the whole sample (*r* = 0.576, *p* = 0.008). Specifically, high levels of brand engagement correlate with high levels of purchase intention, regardless of the type of advertising viewed (Figure 4).

Finally, the correlational analysis highlighted no significant correlations between brain activity and purchase intention, both considering the two experimental groups individually (COVID-19; non-COVID-19) and considering the whole sample. In addition, no significant correlations were found between brain activity and brand engagement, both considering the two groups individually (COVID; non-COVID) and considering the whole sample. The absence of statistically significant correlations was found in both O2Hb and HHb indices.

## 4. Discussion

In the present study, emotional appeals relating (or not) to COVID-19 were used to determine the impact of emotional cues on the brain activity, on the one hand, and on the implicit attitude toward the brand and the intention to purchase from the brand on the other. The findings revealed increased recruitment of PFC during the viewing of COVID-19-related commercials compared to ads unrelated to COVID-19, providing support for our first hypothesis. Indeed, a substantial body of literature shows that several cortical areas within the PFC contribute to the neural network that controls attention shifting towards emotional elements and the processing of emotional information, such as the anterior cingulate cortex (ACC), the orbitofrontal cortex (OFC), and the dorsolateral (DLPFC) and medial prefrontal cortices (mPFC) [57,58,59,60]. This finding might imply that the message conveyed by COVID-19-related advertisements is effective in prompting a deeper elaboration of the affective information. However negative the emotional content may be, developing a play of references on the pandemic (a major disrupting event we all are affected by and we all can relate to) may prove to be a powerful strategy to gain consumers’ attention and emotional engagement.

In addition, the study highlighted the greater recruitment of the left DLPFC with respect to the left BA 45 (Broca’s pars triangularis) in both COVID-19 and non-COVID-19 advertising conditions, but the former appeared to enhance the effect the most. While the left BA 45 is largely believed to be selectively recruited by the elaboration of semantic materials, both written and spoken [61], the DLPFC is involved in emotion regulation processes and emotional reactivity [62,63,64]. In addition, as discussed before, the left-lateralization of DLPFC activity is assumed to mirror an individual’s approach motivational tendencies elicited by specific stimuli irrespective of the emotional valence of the stimuli themselves [22,42,65]. Based on these premises, a possible explanation for our results is that the advertising may have forcibly directed the viewer’s focus towards the emotional component of the ad, implicitly fostering extensive processing of the affective content and prompting an effort to regulate the emotional responses, while making the analysis of the semantic meaning less relevant. In particular, the viewing of COVID-19 pandemic cues may have further enhanced the degree of emotional engagement, drawing the viewer’s attention and determining greater activation of the left DLPFC. As a matter of fact, numerous studies revealed that emotional stimuli typically qualified by negative valence, but linked to active response (such as angry faces), they engender an increase in left PFC activity, which is indicative of the approach motivational system [23]. Specifically, evidence from the consumer neuroscience field highlighted that even ugly advertising scenes, eliciting negative emotions, can prompt left-hemispheric predominance as long as the scenes are perceived as entertaining (i.e., prompting approach tendencies [66]).

As far as the purchase intention is concerned, the absence of any meaningful correlation between the willingness to buy from the brand and the neurovascular modifications within the PFC was ascertained. Since, as discussed, the modifications found in the PFC neural pattern of activity may be indicative of a greater emotional engagement following the COVID-19-related appeals and of a more favorable disposition towards the brand [25], this result suggests that the shifting of the implicit evaluation of the brand after COVID-19-related ads is not followed by an equally tangible adjustment of the explicit purchase intention. Hence, the differential effect of the advertising type (i.e., COVID-19-related or unrelated) appears to be somehow effective only in the modulation of the neurovascular response, possibly shaping the emotional response and the latent attitude towards the brand. Conversely, the intention to purchase does not seem to be sensitive to the emotional information referring to the pandemic displayed during the ads.

Additionally, a significant positive correlation was found between the purchase intention and the brand engagement when considering the entire group of respondents, putting forward the possibility that the prior level of engagement with the brand tends to influence the will to buy from the brand over and above the effect of the experimental manipulation, namely, irrespective of whether the participants saw the COVID-19-related appeals or not. The absence of significant correlations within the two distinct groups, in this case, could be due to the limited sample size of the two groups considered separately. However, considering that in the non-COVID-19 group a marginally significant positive correlation was revealed, it may be possible that purchase intention scores were more considerably affected by the prior engagement with the brand than by the experimental manipulation with neutral advertisements. Conversely, as PI scores within the COVID-19 group were not significantly affected by prior levels of brand engagement, it is possible that COVID-19 contents could be more effective in overwriting the influence of brand preference, affecting the willingness to buy from the brand over the personal preference for the brand.

Regarding the positive correlation found within the whole sample, it may be worth bearing in mind that the brand engagement in self-concept assessed in the present study (BESC) [49] represents a measure of how strongly a brand is perceived to be part of the self-concept. The self-concept is referred to as a mental schema, that is, a knowledge structure encompassing any aspects of the self, which is stored in long-term memory and, thus, is stable to a considerable extent [67]. As a consequence, any aspect comprised in such mental representation of the self—including the brand engagement with brands that are important for the self—could be hardly affected by a one-off experimental manipulation.

Moreover, strong brand engagement is generally associated with high levels of brand loyalty [49]. For this reason, it is possible that the emotional appeals referring to the pandemic scenario, however impactful and effective in modulating the affective and implicit response, did not succeed in generating detectable changes at the explicit level, failing to modify the participants’ conscious intention to purchase from the brand. The literature has indeed provided an abundance of evidence in support of the dissociation between explicit and implicit cognitive processes that make up the consumer’s decision making [68,69]. In fact, explicit measures (i.e., self-reports, interviews, etc.), which require the consumer to put into words her opinion about a brand or a product, are not the most suitable tools to capture and represent implicit evaluations and unconscious processes which—even though they occur outside of awareness—still have a decisive influence on the consumer’s preference and, eventually, on the choice of purchase.

This last consideration highlights the first of the limitations that must be taken into account for the present study. As only explicit judgments were collected to measure the purchase intention, the study may have failed to uncover eventual modifications of the implicit attitude toward the brand. Adding a behavioral measure, such as the Implicit Association Test (IAT) [70], may provide substantial support to the data and clarify whether the modifications detected in the neural correlates could find a consistent effect in the behavioral response, which is governed by (and reflects) automatically activated evaluations. Relatedly, another limitation is that the emotional reactions were exclusively examined via neurophysiological indicators, with explicit emotional judgments being somehow overlooked. Moreover, the PFC was the only brain area taken into consideration for the analysis of the neural markers of the emotional response. However, in the light of the multicomponent nature of emotions, it is well-established that emotion processing involves extensive networks of areas that are not restricted to the PFC and whose contribution interoperates with the decision-making process [71,72]. The future understanding of the emotional processes implicated in consumer decision making may benefit from a more comprehensive examination of the neural areas involved in emotion processing, as well as from the use of additional neurophysiological measures that are currently acknowledged as accurate indicators of the emotional response. For instance, electroencephalography (EEG) has widely shown its valuable contribution in the practice of affective computing, including in consumer neuroscience studies [26], and has recently proved the potential of its combined use with fNIRS [73,74]. In addition, autonomic indices, such as electrodermal activity and heart rate, could provide supplementary insights into the role of other emotional response components (such as physiological arousal) on consumer decisions [58]. Moreover, the strength of the statistical analysis in the present study may have been mitigated by the small size of the recruited sample. Future research could aim at reproducing the study by relying on the evidence provided by a power analysis for the recruitment of a larger sample to confirm the soundness of the findings. A final consideration is that the experiment was carried out one year after the outbreak of COVID-19 pandemic, when the state of emergency was still in place in Italy. The significant effect found for the COVID-19-related communication thus may be limited in time, as it may lose strength as the memories of the pandemic become more distant in time.

## 5. Conclusions

In conclusion, despite its limits, the current study sought to contribute to the understanding of the role of emotions conveyed by commercials on the consumer response. Specifically, the study has investigated the effect of highly emotional appeals tied to COVID-19 information on consumer’s emotional reactions and purchase intention. Our findings suggest that exploiting the emotional potential of a hot topic—such as the COVID-19 pandemic—might be an inefficient strategy to affect consumer behavioral intentions, at least at an explicit level. However, the neurophysiological findings suggest that COVID-19-related advertising might enhance the consumers’ emotional reaction, engaging their attention to a higher extent and perhaps shifting their covert behavioral dispositions towards a more favorable evaluation of the brand.

## Data Availability

The datasets used and/or analyzed during the current study are available from the corresponding author on reasonable request.
